# Adrenaline use is associated with excess organ injury and mortality in cardiogenic shock: facts and fiction

**DOI:** 10.1186/s13054-016-1460-9

**Published:** 2016-09-28

**Authors:** Shou-Yin Jiang, Ye-Hua Shen, Xiao-Gang Zhao

**Affiliations:** 1 Department of Emergency Medicine, Second Affiliated Hospital, Zhejiang University School of Medicine; Research Institute of Emergency Medicine, Zhejiang University, 88 Jiefang Road, Hangzhou, 310009 China; 2 Department of Radiology, The Children’s Hospital, Zhejiang University School of Medicine, Hangzhou, 310009 China

We read with great interest the article by Tarvasmäki et al. [1], who observed the relationship between vasopressor/inotrope use and outcome in patients with cardiac shock (CS). Their results indicate that use of adrenaline in CS is associated with increased 90-day mortality and marked worsening of cardiac and renal biomarkers during the first few days. The study underscores the need for randomized controlled trials of adrenaline versus noradrenaline in CS.

However, we have to raise a serious question regarding the undetailed subgroup of patients with cardiac arrest (CA) because we consider that those CA patients should have been excluded from this study or at least should be analyzed in the form of a subgroup. According to their results, 39 % of patients received adrenaline prior to inclusion because of CA. After inclusion, 21 % of patients received adrenaline. We doubt whether the “21 %” were mainly those who suffered from CA. This is of vital importance because it may cause an obvious bias. This is true when we review their data. Their Table 1 explicitly indicates that patients treated with adrenaline had more severe complications with higher proportions of confusion, oliguria, and hyperlactacidemia. However, a large number of CA patients can also present with confusion, oliguria, and hyperlactacidemia if they experience a prolonged low-flow or no-flow state. Therefore, it should be realized that the CA patients were inappropriately included.

Adrenaline use is associated with higher oxygen requirements of the heart [2]. The very recent European and American guidelines for the diagnosis and treatment of acute heart failure do not recommend routine use of adrenaline in CS unless patients have persistent hypotension despite adequate cardiac filling pressures and the use of other vasoactive agents and resuscitation [3, 4]. The use of noradrenaline has been prevalent in the management of critically ill patients and has been given high grade recommendations in guidelines. Thus, we do not think further clinical trials are required to compare adrenaline and noradrenaline for the treatment of CS.

## Abbreviations

CA: Cardiac arrest
CS: Cardiac shock
CS: Cardiogenic shock
