# Supplementary material for: Adrenaline use is associated with excess organ injury and mortality in cardiogenic shock: facts and fiction
Source: Crit Care. 2016 Sep 28;20:292. doi: 10.1186/s13054-016-1460-9 (PMC5039829; doi:10.1186/s13054-016-1460-9)
Supplement: Related texts

# Reply: Authors’ response

**Authors:** Tuukka Tarvasmäki, Johan Lassus, Alexandre Mebazaa, Veli-Pekka Harjola

We thank Zhao and colleagues for their interest in our paper. We agree that showing the results of survival analyses excluding resuscitated patients may be of additional value. Hence, in the subgroup of patients that were not resuscitated, the adjusted 90-day mortality odds ratio for adrenaline (versus other vasopressors) was 6.5 (95 % confidence interval 1.5–27.2, *p* = 0.01). Figure S2 (Additional file 5) in our original paper [1] confirms the detrimental evolution of cardiac and renal biomarkers in patients treated with adrenaline versus other vasopressors in the subgroup of non-resuscitated patients.

While subgroup analyses in the non-resuscitated patients consistently showed that adrenaline was associated with detrimental effects on outcome, we prefer to present the results for the whole cohort—not specifically excluding patients who had cardiac arrest—as resuscitated patients are part of our daily practice. Indeed, a significant proportion of patients (28 % in the CardShock study) with cardiogenic shock are resuscitated.

We would also take the opportunity to clarify the numbers regarding the frequency of adrenaline use. Of all patients included in the study, 21 % (46/216) were treated with adrenaline, defined as the use of continuous infusion (not bolus injection related to, e.g., resuscitation). However, less than half of the patients treated with continuous adrenaline, 39 % (18/46), were resuscitated prior to study inclusion. On the other hand, 70 % of resuscitated patients were not treated later with continuous adrenaline.

It is true that confusion, oliguria, and elevated lactate levels are very common after cardiac arrest, reflecting inadequate organ perfusion also in this scenario. Nevertheless, signs of hypoperfusion are clinical landmark signs of cardiogenic shock, with or without prior resuscitation.

The recently published European guidelines [3] recommend rather restrictive use of adrenaline in the setting of cardiogenic shock, but the level of evidence is low. The latter might explain the surprisingly common use of adrenaline. Moreover, there are no large randomized studies supporting the recommendation to prefer noradrenaline over adrenaline in CS. Altogether, we still believe that there is room for a pivotal trial to identify the most efficient drug regimen with a favorable safety profile in CS.
